# Successful Discontinuation of Infliximab in a Refractory Case of Vasculo-Behçet Disease

**DOI:** 10.1155/2016/1467583

**Published:** 2016-03-13

**Authors:** Akihiro Nakamura, Tomoya Miyamura, Eiichi Suematsu

**Affiliations:** ^1^Department of Internal Medicine and Rheumatology, Clinical Research Institute, National Hospital Organization, Kyushu Medical Center, Fukuoka 810-8563, Japan; ^2^Department of Genetics and Development, Krembil Research Institute, University Health Network, Toronto, ON, Canada M5T 2S8

## Abstract

Reports have shown that antitumor necrosis factor alpha (anti-TNF-*α*) agents including infliximab (IFX) can dramatically suppress the disease activity of refractory vasculo-Behçet disease (vasculo-BD). However, it is completely unknown whether we can discontinue anti-TNF-*α* agents under clinical remission. A 31-year-old patient with vasculo-BD was initially treated with a high dose of steroid and intravenous cyclophosphamide therapy. Six months later, however, the disease recurred. IFX was administered and immediately the disease activity was reduced. Fortunately, we could discontinue IFX after 18-month remission and no recurrence has been observed. Based on previous reports and our patient, all patients who could discontinue IFX sustained clinical remission for at least one year, continued taking immunosuppressive agents such as methotrexate and azathioprine, and had vascular involvements only in non-life-threatening major vessels such as leg or arm arteries/veins. This is a report suggesting the possibility of discontinuation of IFX in vasculo-BD.

## 1. Introduction

Vasculo-Behçet disease (vasculo-BD) is one of the most severe and life-threatening facets of BD which predominantly appears in large blood vessels [1]. Furthermore, unpredictable recurrence is not uncommon even under strict immunosuppressive treatment. However, since there are no control studies for the management of vasculo-BD, specific guidelines and recommendations for treatments are unavailable excluding the usage of strong immunosuppressive agents [2]. This deficiency of strong evidence leads to ambiguity and difficulty when determining therapeutic strategies.

Recent reports reveal inhibition of tumor necrosis factor alpha (TNF-*α*) has dramatic efficacy for the successful treatment of various types of BD, including vasculo-BD, through controlling inflammation [3–6]. In Japan, usage of infliximab (IFX), a chimeric mouse-human anti-TNF-*α* monoclonal antibody, has recently been covered by national health insurance in refractory cases of specific types of BD including entero-BD, neuro-BD, and vasculo-BD. However, it is completely unknown whether we can discontinue anti-TNF-*α* agents for patients with vasculo-BD under clinical remission. We herein report a suggestive case of successful discontinued IFX treatment and sustained long-term remission in a patient with refractory vasculo-BD.

## 2. Case Presentation

**Figure 1:**
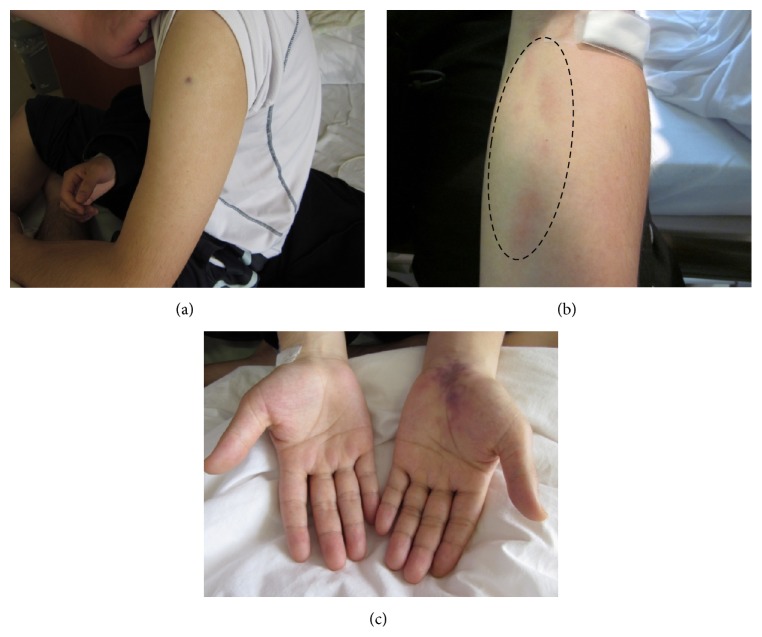
Clinical manifestations on admission. Physical examination identified swollen left upper arm (a), erythema nodosum on the left forearm (b), and purpura on the left wrist joint (c), suggesting a deficiency of blood supply on the left arm.

A 31-year-old previously healthy male was admitted for evaluation for persistent low grade fever and gradual onset of swelling and claudication on his left arm. Further detailed medical history revealed that the patient had a history of recurrent oral aphthae which started 7 years ago. Physical examination on admission determined swelling on his left upper arm, erythema nodosum on his left forearm, and purpura on his left wrist indicating insufficient blood supply (Figures 1(a)–1(c)). Left radial artery pulse was palpable and Allen's test was negative. Joint tenderness was also identified on bilateral knee and ankle joints, suggesting the existence of arthritis. Laboratory studies showed leukocytosis (14,200/*μ*L) with 88.0% of neutrophil, increased levels of serum inflammatory markers such as C-reactive protein (CRP) (12.4 mg/dL), and erythrocyte sedimentation rate (ESR) (50 mm/h). Normal results of protein C, protein S, antithrombin, activated partial thromboplastin time (aPTT), anticardiolipin antibody, anti-*β*2GPI antibody, and lupus anticoagulants excluded the possibility of prothrombotic disorders and antiphospholipid syndrome. Antineutrophil cytoplasmic antibodies (ANCA), including proteinase 3- (PR3-) ANCA and myeloperoxidase- (MPO-) ANCA, were negative and hepatitis B virus (HBV) antigen (HBs antigen) and antibody (HBs antibody) which suggest the possibility of polyarteritis nodosa were also not detected. Result of HLA-B51, a well-known allele as BD preposition, was positive. Computed Tomography (CT) demonstrated inflammation of perivascular tissue around left axial artery and CT Angiography (CTA) clearly showed left brachial and radial artery stenosis, suggesting the diagnosis of arteritis from left axial to radial artery. According to the criteria for BD from Ministry of Health, Labor and Welfare in Japan, the patient was diagnosed with BD, specifically vasculo-BD based on prominent vascular manifestations.

Intravenous methylprednisolone (mPSL) (1000 mg/day for consequent three days) and cyclophosphamide (IVCY) pulse therapy (1000 mg/month) were initiated followed by oral prednisolone (PSL) (60 mg/day) according to the European League Against Rheumatism (EULAR) recommendations for the management of BD [2]. Anticoagulation therapies such as anticoagulants, antiplatelet, or antifibrinolytic agents were not considered based on no control studies as well as no evidence of benefit from them as stated in EULAR recommendations [2]. After the immunosuppressive therapy was initiated, clinical symptoms seemed to gradually subside.

**Figure 2:**
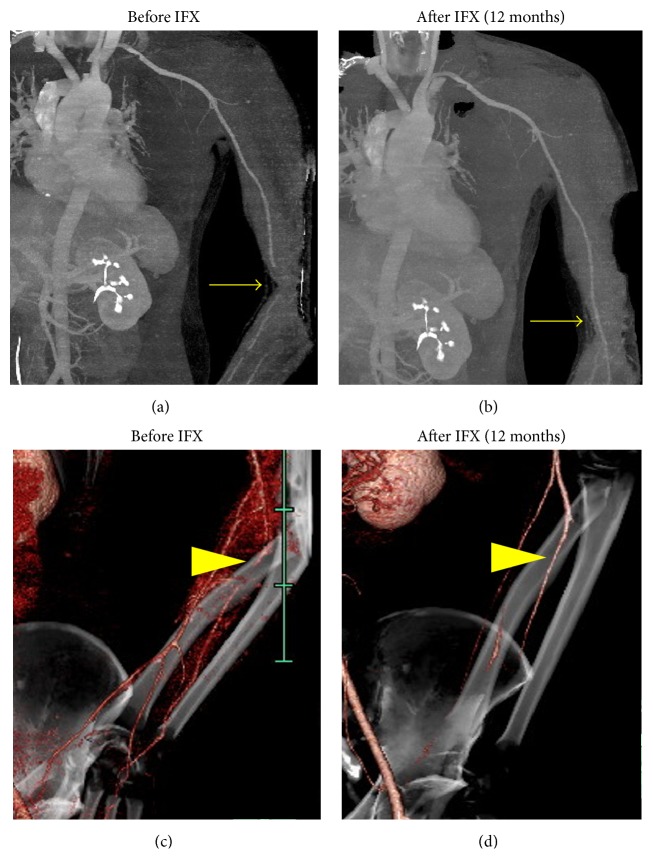
Computed Tomography Angiography showed severe stenosis of left brachial (a) and radial (c) arteries before infliximab (IFX) induction. After IFX therapy, the artery stenosis was immediately resolved and sustained clinical remission ((b) and (d) at 12 months since IFX was introduced).

**Figure 3:**
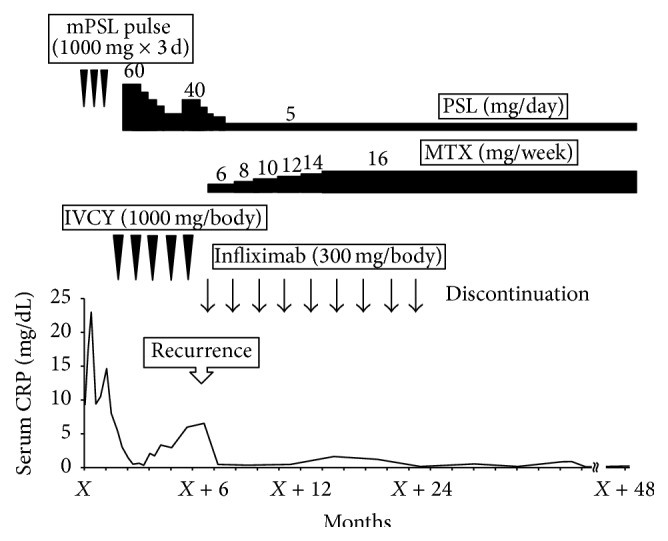
Patient's clinical time course.* mPSL* methylprednisolone,* PSL* prednisolone,* IVCY* intravenous cyclophosphamide, and* MTX* methotrexate.

Six months later, however, left arm pain and claudication recurred and levels of serum inflammatory markers such as CRP and ESR were elevated. At this time, the fifth time of IVCY had ended and the patient was taking 20 mg of PSL. After confirming the diagnosis with recurrent vasculo-BD based on severe stenosis of left brachial and radial artery on CTA (Figures 2(a) and 2(c)), IFX was started (5 mg/kg, 0, 2, and 6 weeks followed by every 8 weeks) in combination with oral methotrexate (MTX) (16 mg/week). Since the induction of IFX and MTX treatment, the symptoms including pain were alleviated within four weeks along with the rapid improvement of serum inflammatory markers. Additionally, the artery stenosis assessed by a repeated CTA was dramatically resolved (Figures 2(b) and 2(d)). Since then, the patient sustained a good response to IFX resulting in clinical remission (no clinical symptoms and normal ranges of serum inflammatory markers) with no remarkable drug side effects. Therefore, we planned to continue IFX. However, IFX was discontinued after 18 months by the patient due to the medical cost. Considering (i) sustained clinical remission of 18 months, (ii) limited artery involvement (only left brachial and radial artery), and (iii) continuation of taking MTX and PSL, we decided to discontinue IFX. Fortunately, no recurrence has been observed for 24 months, with PSL (5 mg/day) and MTX (16 mg/week) as a maintenance therapy (Figure 3).

## 3. Discussion

Vasculo-BD is one of the most severe conditions that predominantly affect young men [1]. Given inflammation of all sizes (small, medium, and large) of blood vessels (both venous and arteries), most clinical manifestations observed in BD can be explained by vasculitis. Furthermore, even in patients without obvious vascular involvements of BD, endothelial and microvascular functions are compromised the same as patients with vascular involvements [7]. However, when specifically describing the term of vasculo-BD, involvements of large blood vessels predominantly appear among clinical manifestations. Superficial venous thrombophlebitis (SVT) or deep venous thrombosis (DVT) has been reported as the most common manifestations in vasculo-BD [8], but arterial lesions including pulmonary artery aneurysms that can result in high mortality are also observed, suggesting early diagnosis and rapid induction of appropriate treatments are crucial. Conventional therapies including glucocorticoid and other immunosuppressive agents such as cyclophosphamide, azathioprine (AZA), and cyclosporine A may suppress the inflammation in both affected venous and arteries, resulting in downregulation of disease activity [9]. However, unpredictable recurrence of vascular events is not uncommon. Additionally, no specific biomarkers for BD hamper the judgment of opportune time for tapering the dose of glucocorticoid and immunosuppressive agents, leading to multiple burdensome side effects.

Usage of TNF-*α* inhibitors for BD has firstly been established as an optimal drug in cases of ocular BD [10] and then the application has been extended to other types of BD, especially in refractory cases showing resistance to steroid and other conventional immunosuppressant therapies. Although the exact mechanism of vasculo-BD is not fully understood, since immunological cells including neutrophils and mononuclear cells (predominantly CD3+CD4+ T lymphocytes and NK cells) infiltrate into the media and adventitia of arterial wall, dysregulated expression of proinflammatory mediators such as TNF-*α*, interleukin- (IL-) 1*β*, IL-6, and IL-17 may be involved in the pathogenesis of vasculo-BD [11]. TNF-*α* produced primarily by cells of the macrophage-monocyte lineage showed biologic effects including adhesion molecule expression, synthesis of proinflammatory cytokines and chemokines, activation of immune system cells, and inhibition of regulatory T-cells; thus, it may directly participate in vascular inflammation as well as endothelial cell damage [7]. Previous reports suggested that anti-TNF-*α* agents show not only the dramatic efficacy on vasculo-BD but also their safety throughout the period of treatment [3–6]. Additionally, it has also been reported that switching drugs between anti-TNF-*α* agents are effective in refractory cases of BD [12]. However, it is a crucial problem for any autoimmune diseases including BD whether biologic agents can be discontinued after achievement of clinical remission to minimize drug side effects, complications, and medical costs. Recent studies on rheumatoid arthritis (RA) demonstrate that discontinuation of biologic agents, including anti-TNF-*α* agents, could be feasible for maintaining low disease activity without any additional therapies, suggesting potential of providing clinical benefits for patients with RA [13].

The patient initially seemed to respond to a high dose of steroid therapy followed by IVCY, but vasculo-BD involved in left brachial and radial arteries recurred. We decided to use IFX for this patient based on a number of reports showing the efficacy of IFX for refractory vasculo-BD and thus the disease activity was eventually reduced [3–6]. Furthermore, it is noted that we were fortunate to be able to discontinue IFX after sustaining 18-month remission with no recurrence for 24 months. However, to date there are no indications for cessation of IFX in vasculo-BD.

To our knowledge, the only report published by Adler et al. showed two BD patients with leg artery and venous thrombosis or iliac vein thrombosis successfully discontinuing IFX after sustaining clinical remission for 3 years and 13 months, respectively. Similar to our patient, MTX or AZA was continued as a maintenance therapy after IFX discontinuation. Although the efficacy of MTX on vasculo-BD has not been established, the combination therapy with anti-TNF-*α* agents seemed to be effective in other BD-related vasculitis including retinal and entero-vasculitis [14], suggesting MTX can also be a reasonable immunosuppressive agent for vasculo-BD. It might be possible that our patient could be managed only by MTX at the recurrence, but it is well-known that the anti-inflammatory effect of MTX appears relatively slow compared to anti-TNF-*α* agents. Furthermore, in Japan the starting dose of MTX is usually low dose (from 6 to 8 mg/week) with concern about side effects such as gastrointestinal symptoms, which means only using low dose MTX would not be enough treatment as a rapid induction therapy for patients with refractory vasculo-BD.

While it is impossible to determine the criteria for IFX discontinuation based only on these patients, we can still identify some tendencies. All patients sustained clinical remission for at least one year, continued taking immunosuppressive agents such as MTX and AZA, and had vascular involvements only in non-life-threatening major vessels such as leg or arm arteries/veins.

Note that this report does not recommend discontinuing IFX in vasculo-BD. Magro-Checa et al. reported that life-threatening vasculo-BD emerged right after discontinuation of IFX even after 3 years of clinical remission [15]. Moreover, in the clinical trial for uveitis in BD, ocular attacks recurred in all patients after interruption of the therapy [10], presumably because concomitant use of immunosuppressive agents was prohibited in the trial. According to these reports, very careful judgement is necessary in deciding whether IFX can be discontinued or not; otherwise disease recurrence resulting in severe condition may occur.

Conclusively, this is a report to suggest the possibility of discontinuing IFX for vasculo-BD to date. Although further investigation and large cohort studies are necessary, this report could provide some clues about the discontinuation to prevent the implications of unnecessary treatment such as bothersome side effects and numerous medical costs.

## Additional Points

It is controversial how to discontinue biologic agents in any diseases, because postdiscontinuation relapses are common. The consensus has not been shown even in RA in which a large number of patients receive biologics including IFX. In the clinical trial for uveitis in BD, ocular attacks recurred in all patients after interruption of the therapy [10], presumably because concomitant use of immunosuppressive agents was prohibited in the trial. Moreover, a recent case report showed that vascular involvement developed 4 months after discontinuation of IFX which maintained remission for 3 years [15], suggesting that decision making of the discontinuation is maybe more difficult in multiorgan diseases such as BD than single-organ diseases. However, this case report demonstrated the possibility of discontinuation of IFX in vasculo-BD. Based on previous reports and our patient, all patients who could discontinue IFX sustained clinical remission for at least one year, continued taking immunosuppressive agents such as MTX and AZA, and had vascular involvements only in non-life-threatening major vessels such as leg or arm arteries/veins. We believe this suggestive case would provide us with some clues about discontinuation of biologic agents including anti-TNF-*α* agents, resulting in keeping patients from huge medical costs, drug side effects, and complications.
